# Skeletal Effects of Bone-Targeted TGFbeta Inhibition in a Mouse Model of Duchenne Muscular Dystrophy

**DOI:** 10.3390/life11080791

**Published:** 2021-08-05

**Authors:** Juliana Marulanda, Iris Boraschi-Diaz, Pierre Beauparlant, Philippe Crine, Frank Rauch

**Affiliations:** 1 Shriners Hospital for Children-Canada, Montreal, QC H4A 0A9, Canada; juliana.marulanda@mail.mcgill.ca (J.M.); iris.boraschidiaz@mail.mcgill.ca (I.B.-D.); 2 Department of Pediatrics, McGill University, Montreal, QC H3A 0G4, Canada; 3 PreciThera Inc., Montreal, QC H3A 2R7, Canada; pbeauparlant@precithera.com (P.B.); philippe@crine.onmicrosoft.com (P.C.)

**Keywords:** bone remodeling, Duchenne muscular dystrophy, fractures, transforming growth factor beta

## Abstract

Duchenne muscular dystrophy (DMD) is a severe progressive muscle disease that is frequently associated with secondary osteoporosis. Previous studies have shown that TGFbeta inactivating antibody improves the muscle phenotype in *mdx* mice, a model of DMD. In the present study, we assessed the skeletal effects of treatment with a bone-targeted TGFbeta antibody (PCT-011) in *mdx* mice. Micro-computed tomography showed that 8 weeks of intraperitoneal administration of PCT-011 (10 mg per kg body mass, 3 times per week) was associated with more than twofold higher trabecular bone volume at the distal femur, which was explained by a higher trabecular number. At the femoral midshaft, PCT-011 exposure increased cortical thickness but did not significantly affect the results of three-point bending tests. Histomorphometric analyses of the lumbar vertebra 4 showed that PCT-011 treatment led to a lower bone formation rate. In conclusion, treatment with the TGFbeta antibody PCT-011 had a positive effect on bone development in *mdx* mice. Inhibiting TGFbeta activity thus appears to be a promising approach to treat bone fragility in the context of DMD.

## 1. Introduction

Duchenne muscular dystrophy (DMD) is an x-linked recessive disorder that is caused by loss-of-function mutations in the *DMD* gene [1]. The resulting lack of dystrophin protein in muscle cells leads to progressive muscle disease. As correction of the underlying gene defect is not feasible at present, current treatment approaches aim at symptomatic improvements. Oral glucocorticoids are widely used to slow down the progression of muscle degeneration [1].

One of the downstream consequences of *DMD* mutations is increased chronic activation of transforming growth factor beta (TGFbeta) signaling, which plays a central role in the cycles of degeneration and regeneration in skeletal muscle that eventually lead to the replacement of muscle by fat and fibrotic tissue [2,3]. TGFbeta promotes the differentiation of myogenic cells into fibrotic cells [4]. Genetic variants that facilitate TGFbeta release from the extracellular matrix are associated with earlier ambulatory loss and accelerated worsening of respiratory muscle function in boys with DMD [3,5]. Conversely, inhibiting TGFbeta activity has beneficial effects in dystrophic muscle, by enhancing differentiation and fusion of precursor satellite cells [6]. In the *mdx* mouse model of DMD, treatment with TGFbeta neutralizing antibody increased grip strength and improved respiratory function [6,7]. Thus, therapeutic approaches to inhibit TGFbeta can improve muscle dysfunction in *mdx* mice.

Apart from the primary muscle defect, the bone disease is a frequent clinical problem in DMD [8,9]. Boys with DMD have multiple risk factors for developing secondary osteoporosis, including muscle weakness, reduced mobility as well as glucocorticoid therapy [10,11,12]. Osteoporosis in boys with DMD leads to fractures of the extremities and vertebral bodies [8,9]. Overall, 30% of boys with DMD have extremity fractures and more than half will eventually have a vertebral fracture [11,12]. Fractures in boys with DMD can lead to premature loss of ambulation and even death [13,14]. Thus, approaches to prevent and treat osteoporosis are an important component of DMD care [8,9].

Drugs from the class of bisphosphonates inhibit bone resorption and are widely used to strengthen bones in many clinical situations, including DMD [8]. Previous studies on boys with DMD have shown that treatment with intravenous bisphosphonates improves back pain caused by vertebral fractures, increases spine bone mineral density, and promotes the reshaping of fractured vertebral bodies [15]. However, some boys with DMD still sustain new vertebral fractures despite receiving bisphosphonate treatment [15]. Better treatment options are thus clearly needed to improve bone health in boys with DMD.

TGFbeta pathways are active in muscle, in addition to having important functions in bone [16]. Mice overexpressing TGFbeta in bone have osteoporosis [17] and mice with genetic TGFbeta inhibition have stronger bones [18]. Similarly, pharmacologic inhibition of TGFbeta with a neutralizing antibody leads to increased bone mass and stronger bones in mice with osteogenesis imperfecta, a disease that is characterized by weak and fragile bones [19].

It is unknown at present whether TGFbeta inhibition has an effect on bone in the context of DMD. In the present study, we, therefore, assessed the skeletal effects of pharmacological TGFbeta inhibition in the *mdx* mouse, a widely used model of DMD. TGFbeta was inhibited using PCT-011, which consists of an antibody that neutralizes all three isoforms of TGFbeta (fresolimumab) to which 10 aspartic acid residues were added at the c-terminal end to target the molecule to the bone. A similar bone-targeting approach has been successful in directing enzyme replacement therapy to the bone in hypophosphatasia, a disorder caused by lack of alkaline phosphatase in bone tissue [20,21]. Results of PCT-011 treatment in *mdx* mice were compared to treatment with prednisolone. We also assessed the effect of treating *mdx* mice simultaneously with prednisolone and PCT-011.

## 2. Materials and Methods

### 2.1. Animals

Male wild type (WT; strain: C57BL/10ScSnJ) and *mdx* mice (strain: C57BL/10ScSn-Dmd*^mdx^*/J) used for this study were purchased from Jackson Laboratory. Animal experiments were performed by Charles River Inc. (Kuopio, Finland). All mice were euthanized at the end of the observation period, and bone and serum samples were shipped to Shriners Hospital for Children in Montreal for analysis. All experiments were approved by the National Animal Experiment Board of Finland and according to the National Institutes of Health (Bethesda, MD, USA) guidelines for the care and use of laboratory animals.

### 2.2. PCT-011 Treatment and Sample Collection

PCT-011 was produced by Evitria (Zurich, Switzerland). The study drug was administered for a duration of 8 weeks, starting at 5 weeks of age. Five groups of mice, with 12 mice each were used for the study. One group consisted of male WT mice receiving vehicle (phosphate-buffered saline, PBS, intraperitoneal injections of 5 mL per kg body mass the injection volume equivalent to the active drug groups, 3 injections per week), the other 4 groups were comprised of male *mdx* mice. The *mdx* mice received either PBS, prednisolone (daily oral dose of 1 mg per kg body mass administered by gavage), PCT-011 (given by intraperitoneal injections of 10 mg per kg body mass, administered 3 times per week, which corresponds to the dose usually used for the similar TGFbeta inactivating antibody 1D11 [19,22]), or a combination of prednisolone and PCT-011 at the indicated doses. Our pilot studies using prednisolone at 5 mg per kg body mass, a widely used dose in studies on mdx mouse muscles [23], showed that this dose consistently led to an increase in trabecular bone mass at the distal femur, which is the opposite of what is observed in boys with DMD receiving glucocorticoids [8]. Therefore, prednisolone was given at a dose of 1 mg per kg body mass, where the paradoxical increase in trabecular bone mass was not observed. A total of 12 mice per group were used.

Mice were euthanized at the end of the intervention period (at the age of 13 weeks). Blood samples were collected at euthanasia by intracardiac puncture, and serum was separated by centrifugation and stored at −80 °C until analysis. Both femurs and tibias were dissected. Tibia length was measured with a digital caliper. Right femurs were used for micro-computed tomography (microCT). Following microCT, the right femurs were subjected to three-point bending tests. These samples were stored at −20 °C in phosphate-buffered saline-soaked gauze until testing.

### 2.3. Grip Strength Measurements

The Animal Grip Strength System (San Diego Instruments, San Diego, CA, USA) was used. Mice were placed on a small mesh that they gripped with the forepaws. The mice were then slowly pulled away from the mesh by the tail until they released the grip. The equipment automatically measured the maximum force of the animal’s grip until release. Five scores were recorded per animal in consecutive sequence, and the average of the three best scores for each mouse was used for the results.

### 2.4. Serum Biochemistry

Markers of bone formation (procollagen type I N-terminal propeptide, PINP; Mouse/Rat PINP, Immunodiagnostic Systems) and of bone resorption (C-telopeptide of collagen type I, CTX; RatLaps, Immunodiagnostic Systems) were quantified by enzyme immunoassays. Serum activity of alkaline phosphatase was determined by the kinetic photometric 2-amino-2-methyl-1-propanol method (McGill Diagnostic Laboratory, Montreal, QC, Canada).

### 2.5. MicroCT

MicroCT of the right femur was performed using a Skyscan 1272 device (Bruker). Scan parameters included a 5 μm voxel size, 0.40-degree increment angle, 3 frames averaged, a 66 kV and 142-mA X-ray source with a 0.5-mm Al filter to reduce beam-hardening artifacts. At the distal femur, trabecular bone was analyzed in a region starting at 0.5 mm proximal of the distal femoral growth plate (to avoid primary spongiosa) and scanning a 1.0 mm section of bone in a proximal direction, with an adaptative threshold of 100–255 To analyze cortical bone at the midshaft femur, scanning was performed starting at 44% of the total femur length from the distal end and scanned for 1 mm proximally. The software derives outer bone diameter, cortical thickness, and the diameter of the bone marrow cavity from cross-sectional areas using a circular bone cross-sectional model. The global threshold used was 62–255. Scans were quantified using the system’s analysis software (Skyscan CT Analyser, Version 1.16.1.0).

### 2.6. Biomechanical Testing

Following microCT scanning, right femurs were loaded to failure in three-point bending using a Materials Testing System Model 5943 (Instron, Norwood, MA, USA). The specimens were thawed one day prior to the test and all muscle tissues were cleaned off. The bone was soaked overnight in phosphate-buffered saline at room temperature until mechanical testing. The fixed distance between the lower supporting bars was 7 mm, with a vertical load cell of 40 N and a displacement rate of 50 μm/s. The anterior mid-diaphysis was loaded under tension, and the tests were analyzed using the system’s analysis software Bluehill (Illinois Tool Works, Version 3.65).

### 2.7. Bone Histomorphometry

A separate study was performed to assess the effect of PCT-011 treatment on histomorphometric parameters of bone metabolism in *mdx* mice. One group of *mdx* mice received intraperitoneal injections of PBS (5 mL per kg body mass, 3 times per week), the other received intraperitoneal injections of PCT-011 (10 mg per kg body mass, 3 times per week) from the age of 4 weeks to the age of 10 weeks. A total of 10 mice were used per group. Each mouse received two intraperitoneal injections of calcein (20 mg per kg body weight) at 5 days and at 2 days before sacrifice to enable histomorphometric analyses of dynamic bone formation parameters.

Lumbar spine specimens were fixed in 10% phosphate-buffered formalin, dehydrated in increasing concentrations of ethanol, and embedded in methylmethacrylate. Undecalcified 6 μm thick sections were cut with a Polycut E microtome (Reichert-Jung, Heidelberg, Germany). The sections were deplastified with ethylene glycol monoethyl acetate to allow for optimal staining. In each sample, two consecutive sections were selected that were stained with Masson Goldner Trichrome for static parameters or mounted unstained for the measurement of dynamic parameters using fluorescence microscopy. Histomorphometric analyses of L4 trabecular bone analyzed the entire trabecular compartment excluding a 50 μm band adjacent to each end plate. Measurements were carried out using a digitizing tablet with Osteomeasure^®^ software (Osteometrics Inc., Atlanta, GA, USA). Nomenclature and abbreviations follow the recommendations of the American Society for Bone and Mineral Research.

### 2.8. Statistical Analyses

All data shown in this report are mean ± SEM. Differences between WT and vehicle-treated *mdx* mice were tested for significance using the unpaired *t*-test. Differences between treatment groups of *mdx* mice were assessed by one-way analysis of variance (ANOVA) with Sidak’s tests for multiple comparisons using GraphPad Prism version 8.3 (GraphPad Software for Windows, San Diego, CA, USA). A *p* Value < 0.05 was considered significant.

## 3. Results

**Figure 1:**
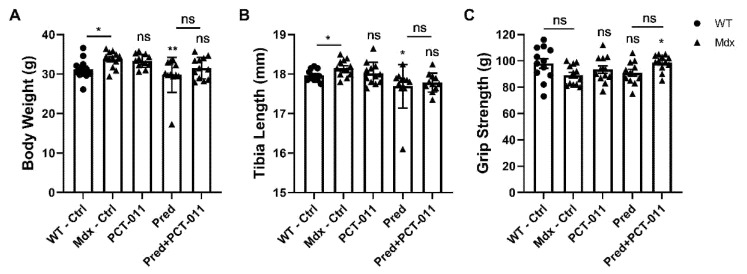
Body weight (**A**), tibia length (**B**), and grip strength (**C**) results. The level of significance for the difference between the vehicle-treated *mdx* group and active-treatment *mdx* groups is indicated above each bar. The level of significance for the difference between vehicle-treated wild-type mice and vehicle-treated *mdx* mice, as well as between prednisolone (Pred) and prednisolone + PCT-011 groups, is shown above the horizontal bars. Levels of significance: * *p* < 0.05, ** *p* < 0.01. *n* = 11–12 samples per group. Data represent mean ± SEM.

Compared to WT mice, vehicle-treated *mdx* mice were heavier and had longer tibias but similar grip strength (Figure 1). Treatment with PCT-011 had no significant effect on any of these measures. Exposure to prednisolone was associated with lower body weight and shorter tibias. The addition of PCT-011 to prednisolone normalized body weight and tibia length and led to significantly higher grip strength.

**Figure 2:**
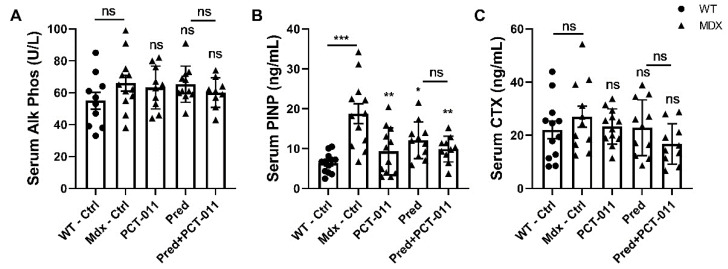
Serum markers of bone and collagen metabolism alkaline phosphatase (**A**), PINP (**B**) and CTX (**C**). The level of significance for the difference between the vehicle-treated *mdx* group and active-treatment *mdx* groups is indicated above each bar. The level of significance for the difference between vehicle-treated wild-type mice and vehicle-treated *mdx* mice, as well as between prednisolone (Pred) and prednisolone + PCT-011 groups is shown above the horizontal bar. Levels of significance: * *p* < 0.05, ** *p* < 0.01, *** *p* < 0.001. *n* = 10–12 samples per group. Data represent mean ± SEM.

Regarding systemic markers of bone and collagen metabolism, serum alkaline phosphatase activity was similar between groups (Figure 2). Serum concentrations of PINP, a marker of collagen type I production, were significantly higher in vehicle-treated *mdx* mice than in WT mice but were suppressed by treatment with PCT-011, prednisolone, or the combination of the two drugs. Serum levels of the bone resorption marker CTX did not vary significantly between groups (Figure 2).

**Figure 3:**
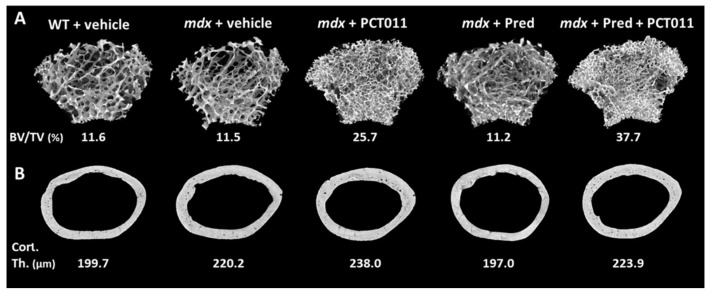
(**A**) Representative microCT images of the distal femur. The trabecular bone volume per tissue volume of each sample is indicated; (**B**) representative microCT images of the femoral midshaft. The cortical thickness of each sample is indicated.

**Figure 4:**
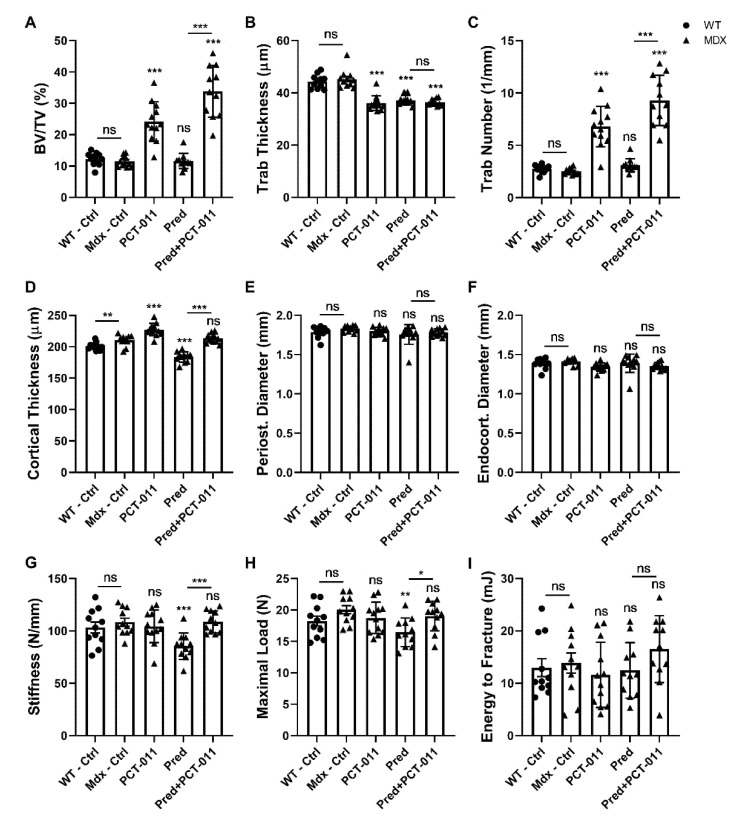
Femur structure and biomechanical properties: (**A**–**C**) trabecular bone assessment by microCT at the distal metaphysis; (**D**–**F**) cortical bone assessment by microCT at the mid-shaft diaphysis; (**G**–**I**) results of three-point bending tests. The level of significance for the difference between the vehicle-treated *mdx* group and active-treatment *mdx* groups is indicated above each bar. The level of significance for the difference between vehicle-treated wild-type mice and vehicle-treated *mdx* mice, as well as between prednisolone (Pred) and prednisolone + PCT-011 groups, is shown above the horizontal bars. Levels of significance: * *p* < 0.05, ** *p* < 0.01, *** *p* < 0.001. *n* = 10–12 samples per group. Data represent mean ± SEM.

MicroCT analyses of trabecular bone at the distal femur showed that vehicle-treated WT and *mdx* mice had similar trabecular microstructure (Figure 3A and Figure 4A–C). Treatment with PCT-011 was associated with more than twofold higher trabecular bone volume per tissue volume despite the lower trabecular thickness because the trabecular number was markedly increased. Prednisolone monotherapy at the dose given in this study had no significant effect on trabecular bone volume per tissue volume. Adding PCT-011 to prednisolone-treated mice increased trabecular bone volume per tissue volume through an increase in trabecular number.

MicroCT analyses at the femoral midshaft (Figure 3B and Figure 4D–F) showed that vehicle-treated *mdx* mice had a thicker cortex than vehicle-treated WT mice. Treatment with PCT-011 further increased cortical thickness, whereas exposure to prednisolone led to lower cortical thickness. These negative consequences of prednisolone on cortical thickness were prevented by simultaneous treatment with PCT-011. None of the interventions had a significant effect on periosteal or endocortical diameter, as these measures varied more widely between samples than the cortical thickness (Figure 4E,F).

Biomechanical analyses of the femur by three-point bending (Figure 4G–I) showed no differences between vehicle-treated *mdx* mice and WT mice. PCT-011 treatment had no significant effect on biomechanical parameters, but exposure to prednisolone was associated with lower bone stiffness and lower maximal load. These negative effects of prednisolone were rescued by the simultaneous application of PCT-011.

**Figure 5:**
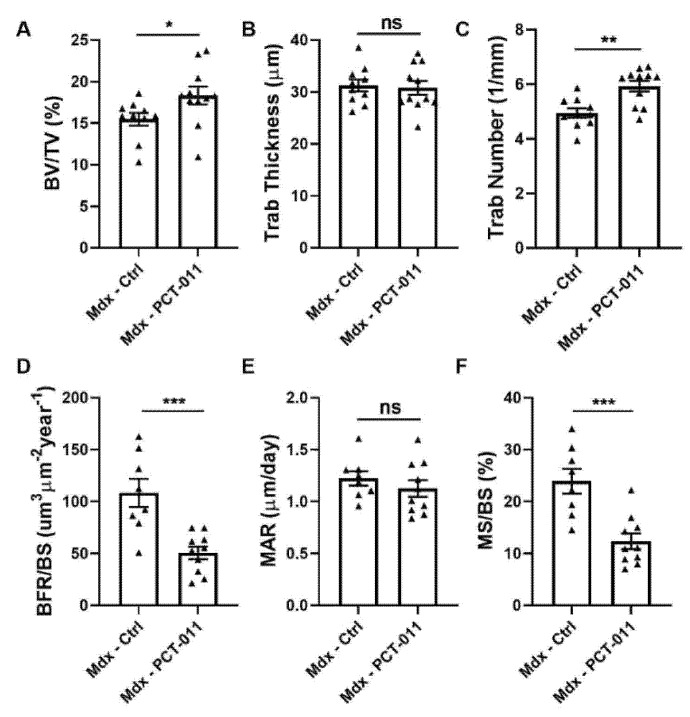
Effect of PCT-011 on trabecular bone in lumbar vertebra 4, as analyzed by histomorphometry: (**A**–**C**) parameters of bone structure. BV/TV: bone volume per tissue volume, Trab Thickness: trabecular thickness, Trab number: trabecular number; (**D**–**F**) dynamic parameters of bone formation. BFR/BS: bone formation rate per bone surface, MAR: mineral apposition rate, MS/BS: mineralizing surface per bone surface. The level of significance for the difference between vehicle-treated *mdx* mice and PCT-011-treated *mdx* mice is shown above the horizontal bar. Levels of significance: triangle: *mdx*, * *p* < 0.05, ** *p* < 0.01, *** *p* < 0.001. *n* = 8–10 samples per group. Data represent mean ± SEM.

In a separate study, we assessed the effects of PCT-011 treatment on the lumbar spine in *mdx* mice (Figure 5). Analyses of trabecular bone structure at lumbar vertebra 4 showed that PCT-011 treatment associated with higher trabecular bone volume per tissue volume and higher trabecular number. Dynamic bone histomorphometry demonstrated that PCT-011 led to a lower bone formation rate. Bone formation rate is calculated as the product of mineralizing surface and mineral apposition rate [24]. Analysis of these components showed that lower bone formation rate after PCT-011 exposure was explained by lower mineralizing surface, whereas mineral apposition rate was not altered by PCT-011.

## 4. Discussion

In this study, we assessed the skeletal effect of the bone-targeted anti-TGFbeta antibody PCT-011 in *mdx* mice. We observed that treatment with PCT-011 alone increased trabecular bone mass and led to thicker cortical bone at the midshaft femur. PCT-011 also prevented the negative effects of prednisolone exposure on bone in *mdx* mice. When PCT-011 was given together with prednisolone, grip strength was increased. A previous study had also found increased grip strength in *mdx* mice treated with the TGFbeta antibody 1D11, which in contrast to PCT-011 is not targeted to bone [7].

The observed effect of PCT-011 treatment on trabecular bone mass is in accordance with previous studies that examined the effect of TGFbeta on bone. Mouse models with increased TGFbeta expression in bone have a loss of trabecula [17]. Conversely, treatment with the systemically active 1D11 anti-TGFbeta antibody increased trabecular bone mass in wild-type mice and in some models of osteogenesis imperfecta [19,22,25]. The present study thus confirms that anti-TGFbeta treatment also has a positive effect on trabecular bone mass in *mdx* mice.

Interestingly, we found that anti-TGFbeta treatment with PCT-011 increased trabecular bone mass mostly by increasing trabecular number rather than through an effect on trabecular thickness, which is similar to our previous observations in WT mice treated with 1D11 antibody [22]. As we administered PCT-011 to mice that were actively growing, these results likely reflect the effect of PCT-011 on trabecular bone development. During growth, new (primary) trabeculae are created by endochondral ossification. Most of the primary trabecula is quickly removed by bone resorption. Treatments that inhibit bone resorption, therefore, prevent the removal of primary trabecula and lead to higher trabecular numbers. The changes in trabecular microstructure, therefore, suggest that PCT-011 inhibits the resorption of newly created trabecula in the primary spongiosa. Similar observations have been made in young children with osteogenesis imperfecta who received intravenous treatment with another inhibitor of bone resorption, pamidronate [26]. In the present study, group differences for the systemic bone resorption marker CTX were not significant, due to the high variability of results. It is also possible that serum CTX levels are influenced by the breakdown of muscle collagen. Muscle collagen is increased and is structurally abnormal in mdx mice [27]; therefore, it might make a bigger contribution to the circulating pool of CTX in mdx than in WT mice.

One interesting observation of the present study is that circulating levels of PINP were significantly higher in vehicle-treated *mdx* mice than in WT mice. PINP is released during the processing of collagen type I, when the N-propeptide is cleaved from the collagen type I triple-helical domain [28]. As bone normally makes the largest contribution to the whole-body synthesis of collagen type I, PINP usually reflects bone formation [28]. However, in *mdx* mice, muscles undergo extensive fibrosis, and this fibrotic tissue is rich in collagen type I [29,30]. It is, therefore, possible that increased serum PINP levels in *mdx* mice reflect the increased production of collagen type I in the muscle tissue. PINP levels were lowered by treatment of *mdx* mice with either prednisolone or PCT-011, which could be explained by the effect of these interventions on slowing muscle fibrosis. Indeed, TGFbeta inhibition using the 1D11 antibody has been shown to decrease the muscle collagen content of mdx mice [7].

PCT-011 treatment also was associated with an increase in cortical thickness at the midshaft femur. Increased cortical thickness could be expected to lead to stronger bones, but three-point bending test results did not change significantly with the PCT-011 treatment. This indicates that the positive treatment effect on bone geometry was not sufficient to exert a detectable effect on biomechanical parameters of long-bone strength.

Similar to previously published pharmacological studies that targeted bone in the *mdx* mouse, we used glucocorticoid exposure as a control intervention. We noted that prednisolone exposure at a dose of 1 mg per kg body weight was associated with lower body weight, shorter tibias, thinner femoral cortices, and decreased maximal load in the biomechanical testing of the femur. These results document the expected detrimental effect of prednisolone on bone growth and development, as was seen in previous studies where *mdx* mice received glucocorticoids [31,32]. Given the widespread use of glucocorticoid treatment in boys with DMD, our finding that the addition of PCT-011 to prednisolone treatment counteracted the negative effect of prednisolone on bone, has potential translational implications.

In conclusion, treatment with the TGFbeta antibody PCT-011 increased trabecular bone mass, led to thicker cortical bone at the midshaft femur, and prevented the negative effects of prednisolone exposure on bone in *mdx* mice. Inhibiting TGFbeta activity thus appears to be a promising approach to treat bone fragility in the context of DMD.
